# Atypical Multifocal Presentation of Congenital Plunging Ranulas: A Radiological Approach to Diagnosis and Treatment

**DOI:** 10.7759/cureus.80027

**Published:** 2025-03-04

**Authors:** Bao H Nguyen, Morgan E Baranek, Craig M Johnson

**Affiliations:** 1 Pediatric Interventional Radiology, University of Central Florida, College of Medicine, Orlando, USA; 2 Pediatric Interventional Radiology, Nemours Children's Hospital, Orlando, USA

**Keywords:** cheek swelling, cystogram, fluoroscopy intervention, head and neck radiology, mri head and neck, oral ranula, plunging ranula, sublingual cyst, sublingual salivary glands, submandibular gland lesions

## Abstract

Congenital plunging ranulas are pseudocystic lesions affecting the floor of the mouth. This case report presents a two-month-old female with an asymptomatic unilateral cheek swelling, diagnosed with a multifocal congenital plunging ranula. Magnetic resonance imaging, ultrasound, and fluoroscopy revealed an atypical, multiseptated submandibular lesion with multifocal sublingual salivary extensions. Needle aspiration of the submandibular lesions yielded mucoid salivary discharge, confirming the diagnosis. The swelling was significantly reduced and the patient continued to achieve all developmental milestones. An appreciation for the complex, multifocal architecture of the lesion on imaging is essential for management because complete excision of the affected salivary glands and extension tracts is required to prevent the recurrence of the ranula.

## Introduction

A ranula is a pseudocystic swelling in the floor of the mouth caused by salivary retention from the sublingual gland. Ranulas are classified as non-plunging, confined to the floor of the mouth, or plunging, which extend into the cervical region [1]. Most ranulas are non-plunging and occur in adults due to trauma, infection, or iatrogenic causes [1,2]. Only 20% occur in children under age 16 years, and of those, just 10% are plunging, with the congenital variant being exceptionally rare [3-5]. Nearly all reported plunging ranulas are unifocal, with a single salivary extension from the sublingual gland [6,7]. Chowdhary et al. described a rare congenital plunging ranula extending from the sublingual to the submandibular space [5]. However, no cases of multifocal congenital plunging ranulas have been reported. Here, we present an unusual case of congenital plunging ranula with multiple sublingual origins and extensions into the submandibular space, creating a complex, multiseptated appearance on imaging.

## Case presentation

**Figure 1:**
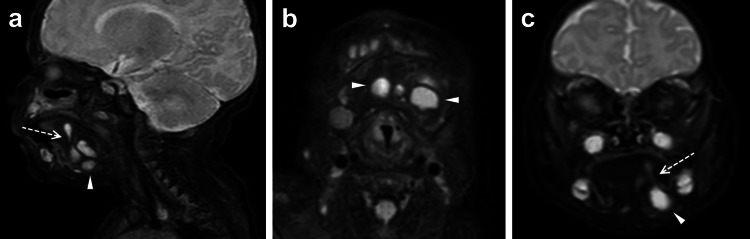
(a) Sagittal, (b) axial, and (c) coronal T2-weighted postcontrast MRI images demonstrated a multiseptated cystic lesion (arrowheads) in the submandibular space. A tail (dashed arrows) extends from the sublingual space and communicates with submandibular space, consistent with a plunging ranula.

A two-month-old female infant presented with a non-erythematous, non-fluctuant, and diffuse swelling of the left cheek, which had gradually increased in size since one month of age. Intraoral examination was unremarkable. The patient was born at term, had no past medical history (no infection, trauma, or previous surgery), and had been feeding well and achieving all developmental milestones. Magnetic resonance imaging (MRI) of the head and neck revealed a multiseptated lesion measuring 2.6 x 2.7 x 2.6 cm in the left submandibular space (Figure 1). 

**Figure 2:**
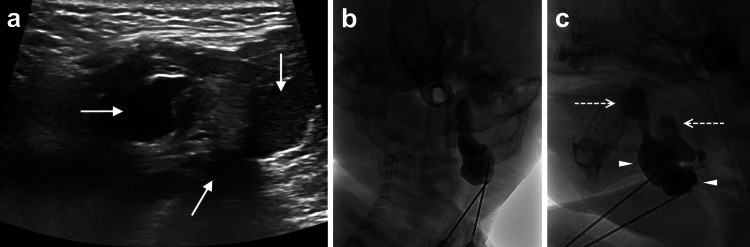
(a) Ultrasound showed three left submandibular lesions (solid arrows), with one lesion displaying increased, heterogenous internal echogenicity compared to two other more hypoechoic lesions. Following aspiration of the lesions, a cystogram, on coronal (b) and sagittal (c) views was performed by injecting fluoroscopic contrast into two of the sites, which demonstrated discrete fluid pockets (arrowheads) with discrete extensions from the left sublingual space (dashed arrows).

Diagnostic aspiration was performed by interventional radiology under general anesthesia. Pre-operative ultrasound revealed three lesions: two homogenous, hypoechoic cystic lesions and one heterogenous lesion with internal hyperechogenicity (Figure 2a). Under sonographic guidance, approximately 1 mL of cloudy mucoid fluids were aspirated from each of the pockets. A cystogram was then performed by injecting fluoroscopic contrast into two of the aspirated sites, revealing discrete fluid pockets at the submandibular level with extensions originating from the left base of the floor mouth, at the sublingual space (Figure 2b, 2c). No contrast extravasation into surrounding tissues was observed, and all contrast was subsequently removed. The postoperative course was uneventful and the patient was discharged without any complications.

In summary, the patient was found to have a multiseptated pseudocystic lesion in the submandibular space with multifocal salivary extensions of sublingual origin. The aspirated fluid was highly viscous, further supporting a diagnosis of a congenital plunging ranula. At six-month follow-up, the swelling was significantly reduced. The patient had no difficulty with feeding and continued to achieve all developmental milestones. She will continue to be monitored by her otolaryngologist.

## Discussion

Traditionally, the diagnosis of ranula has been clinical, especially for the acquired type which has an identifiable etiology such as facial trauma, infection, or iatrogenic injury from dental or oral surgery [7]. The pathogenesis of the congenital variant is unclear but is potentially due to an inherent defect of the sublingual gland and mylohyoid muscle [5,6]. While not observed in this case, intraoral examination of a plunging ranula may reveal a unilateral, painless, blue-tinted swelling in the floor of the mouth [2]. Most cases are asymptomatic though larger lesions can cause compression of the hypoglossal nerve, difficulty eating, or upper airway obstruction [1]. 

**Figure 3:**
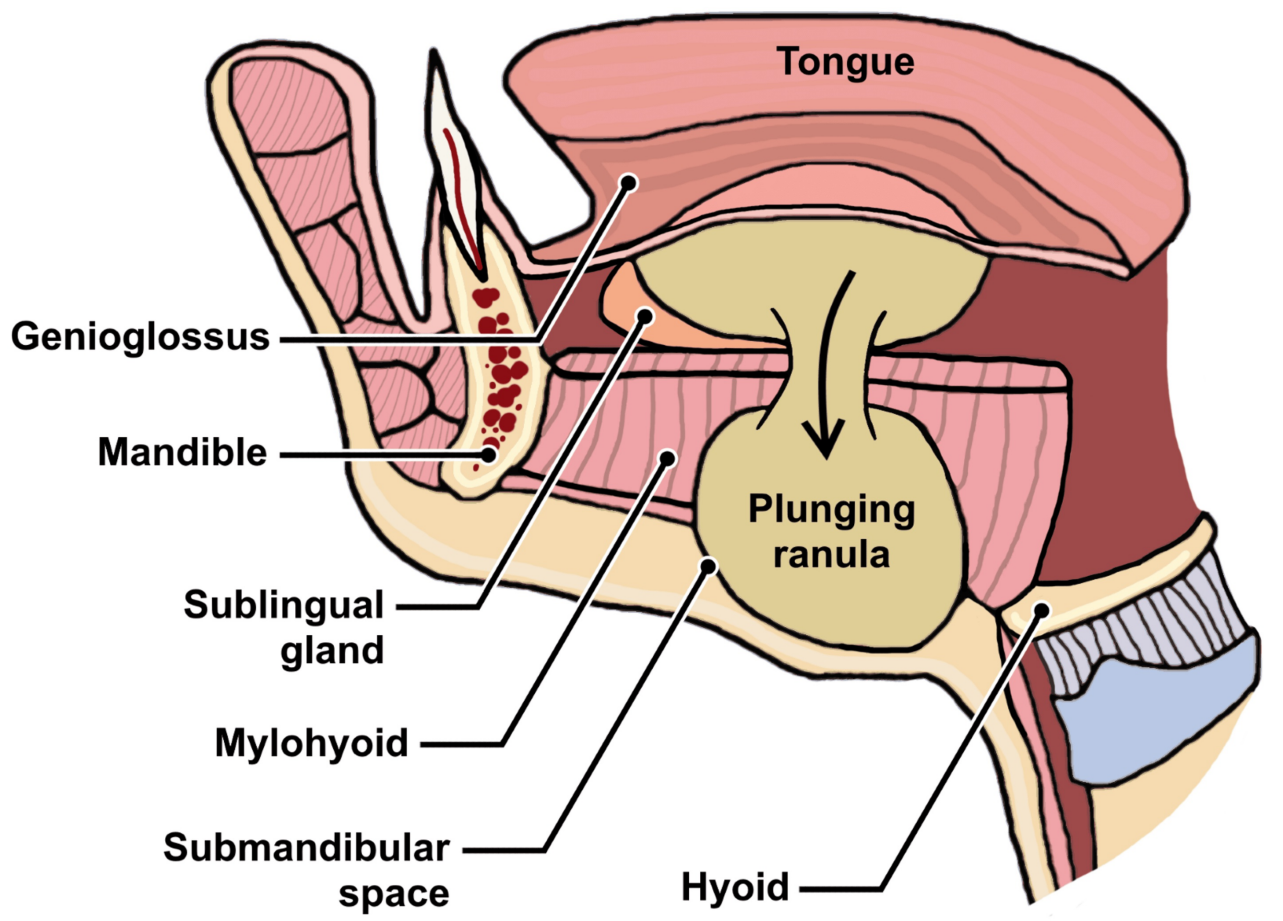
Anatomy of plunging ranulas

Given the ambiguous clinical presentation in this case, MRI and ultrasound were the preferred next steps in the diagnostic workup. Computed tomography was not used due to limited visualization of oral soft tissue pathology and the high radiation dosage to pediatric patients. Notably, the patient's MRI demonstrated the "tail sign," which represents the inferolateral communication of the collapsed sublingual and submandibular spaces over the posterior edge of the mylohyoid muscle (Figure 3). This finding is pathognomic for plunging ranulas [4,8].

Differential diagnoses to consider in evaluating unilateral cheek swelling in the pediatric population include lymphatic malformation, sialocele, infection, abscess, and malignancy. Oral lymphatic malformation frequently presents as soft-tissue swellings, most commonly affecting the tongue, and less commonly the hard or soft palates and buccal mucosa [9]. These lesions often exhibit heterogeneous nodularity, colors, and textures depending on the subtype [9]. Ultrasound may reveal a combination of hypoechoic cystic or hyperechoic hemorrhagic internal components [9]. Sialocele usually presents as a non-tender, mobile swelling following trauma or iatrogenic injury, often appearing as a hypoechoic cyst on ultrasound [9]. Parotid abscesses can cause tender, erythematous, indurated, and fluctuant swellings, sometimes accompanied by fever and leukocytosis, causing inconsolable distress and difficulty feeding in infants [10]. Ultrasound typically shows a complex lesion with variable internal echogenicity due to inflammatory or necrotic debris [10]. Malignancy of the oral cavity is extremely rare in children and is usually associated with neoplastic genetic syndromes or maternal transmission of acquired immunodeficiency [9]. 

Watchful waiting is a reasonable approach for managing small, asymptomatic ranulas. Transoral surgical excision of the lesion and affected sublingual gland is recommended for large or symptomatic ranulas [11,12]. Less invasive techniques, such as incision and drainage, marsupialization, or needle aspiration carry a higher risk of recurrence [10]. Rather than observation, a more invasive diagnostic workup was pursued due to the unique multiseptated and multifocal appearance seen on MRI. For comparison, Mabongo et al. described a unique case of a multiseptated cystic lesion diagnosed as a plunging ranula in a 32-year-old man with HIV [13]. In this case, pre-operative ultrasound demonstrated multiple thin-walled cystic lesions with one of the three pockets containing hyperechoic debris, an atypical finding for ranulas [4,5]. Despite these unusual imaging features, the needle of aspiration of all fluid pockets yielded the same clear-yellow mucoid discharge consistent with salivary gland secretion. In uncertain cases, laboratory analysis for elevated amylase can verify the aspirate's salivary origin [13]. 

This case highlights the advantage of fluoroscopic needle aspiration, as it allows for a simultaneous cystogram to visualize fluid pockets and extension tracts. Understanding the complex architecture of the lesion is crucial for surgical planning, as a complete excision of all the affected regions is necessary to prevent recurrence. However, surgical excision was deferred in this case since the patient remained asymptomatic and continued to meet all developmental milestones. Lastly, this case underscores the importance of multidisciplinary collaboration between surgery and interventional radiology in managing an atypical disease presentation, ensuring the best possible outcome for the patient.

## Conclusions

Congenital plunging ranula is a rare cause of unilateral cheek swelling in pediatric patients and is typically characterized by a single salivary extension from the sublingual gland into the submandibular space. However, as demonstrated in this case, it can also present as a multiseptated lesion with multifocal sublingual origins. When the clinical presentation is unclear, ultrasound and MRI can help characterize the lesion, with the "tail sign" being highly specific for plunging ranula. Fluoroscopic needle aspiration and cystography can further aid in the diagnosis while simultaneously reducing the swelling. This approach may help delay the need for more invasive surgery, particularly in asymptomatic infants who continue to meet developmental milestones.
